# Identification of Allele-Specific RNAi Effectors Targeting Genetic Forms of Parkinson's Disease

**DOI:** 10.1371/journal.pone.0026194

**Published:** 2011-10-21

**Authors:** Christopher R. Sibley, Matthew J. A. Wood

**Affiliations:** 1 Department of Physiology, Anatomy and Genetics, University of Oxford, Oxford, United Kingdom; 2 MRC Laboratory of Molecular Biology, Cambridge, United Kingdom; National Institutes of Health, United States of America

## Abstract

Parkinson's disease (PD) is a progressive neurological disorder affecting an estimated 5–10 million people worldwide. Recent evidence has implicated several genes that directly cause or increase susceptibility to PD. As well as advancing understanding of the genetic aetiology of PD these findings suggest new ways to modify the disease course, in some cases through genetic manipulation. Here we generated a ‘walk-through’ series of RNA Pol III-expressed shRNAs targeting both the α-synuclein A30P and LRRK2 G2019S PD-associated mutations. Allele-specific discrimination of the α-synuclein A30P mutation was achieved with alignments at position 10, 13 and 14 in two model systems, including a heterozygous model mimicking the disease setting, whilst 5′RACE was used to confirm stated alignments. Discrimination of the most common PD-linked LRRK2 G2019S mutation was assessed in hemizygous dual-luciferase assays and showed that alignment of the mutation opposite position 4 of the antisense species produced robust discrimination of alleles at all time points studied. Discrimination at this position was subsequently confirmed using siRNAs, where up to 10-fold discrimination was seen. The results suggest that RNAi-mediated silencing of PD-associated autosomal dominant genes could be a novel therapeutic approach for the treatment of the relevant clinical cases of PD in future.

## Introduction

Genetic mapping of hereditary Parkinson's disease (PD) over the last 12 years has revealed sixteen chromosomal “PARK” loci with linkage to PD. Subsequently a group of nine genes have been identified which are implicated in molecular pathways leading to PD pathogenesis [1]. The precise function and role of each of these genes in non-familial PD remains unclear since only two of these candidates were identified in recent large-scale genome-wide association studies (GWAS) [2], [3]. However, collectively these hereditary cases account for 5–10% of all cases of PD and offer defined therapeutic targets for those patients bearing these genetic mutations.

RNA interference (RNAi) has emerged as a highly promising strategy for conferring sequence-specific silencing of genes-of-interest. The endogenous RNAi pathway involves processing of non-coding RNA sequences, termed primary-microRNAs (pri-miRNAs), into short 21–23 nt single-stranded mature miRNAs that are antisense to targeted transcripts. Post-transcriptional regulation roles for miRNAs have been identified in development and disease [4]. Further, artificial precursors of this RNAi pathway can be generated in order to silence genes-of-interest for research and therapeutic purposes in a sequence-specific manner. Crucially, changes of a single nucleotide can abrogate silencing ability of an RNAi trigger. By designing and screening RNAi triggers perfectly complementary to autosomal dominant mutant alleles at the site of a mutation, a single mismatch will exist between the antisense species and the wild-type allele which can have potential to disrupt silencing ability. In-so-doing the mutant allele can be selectively removed whilst as much as possible of the wild-type allele is retained to carry out endogenous functions. It can be argued that this is the most suitable therapy over a complete silencing of both wild-type and mutant alleles with non-allele specific silencing. Many genes have essential or presently unknown roles which could be eliminated by a complete silencing, potentially leading to damaging effects [5]. In such settings, allele-specific silencing has the obvious advantage that some of the wild-type gene product remains, whilst the pathogenic mutant is eliminated. This allele-specific silencing approach has been exploited to target disease-linked mutations linked to fronto-temporal dementia [6], Alzheimer's disease [7], Huntington's disease [8], amyotrophic lateral sclerosis [9], spino-cerebellar ataxia type 7 [10] and pachyonychia congenital [11].

Autosomal dominant, gain-of-function mutations linked to be PD have also been identified in the α-synuclein and leucine-rich repeat kinase 2 (LRRK2) genes, yet just one study has investigated allele-specific silencing of the A53T α-synuclein mutation [12]. Three pathogenic mutations in α-synuclein lead to increased rates of formation of α-synuclein fibrils and/or intermediate toxic proto-fibrils to suggest a toxic gain-of-function to these mutations [13]–[15]. Over 30 PD-linked mutations have been identified in LRRK2 which has both kinase and GTPase activity [1]. The G2019S mutation, located in the active site of the kinase domain, is the most common PD-associated mutation accounting for 2–8% of all hereditary cases of PD and 0.6–1.6% of PD cases with no obvious signs of familial inheritance implying that it could be a mutational hotspot for sporadic PD [16]. Collectively it suggests that this mutation accounts for ∼1% of all PD cases making it a high-profile therapeutic target.

In this study we experimentally validate and optimise RNAi triggers which selectively target the α-synuclein A30P and LRRK2 G2019S mutations linked to PD. We report successful discrimination of both these mutations using short-hairpin RNAs (shRNAs) with mutations aligned opposite certain, but not all, positions of the antisense species. In both cases, the discriminating abilities of some shRNAs could be further improved by incorporation of secondary mismatches to the wild-type allele. Finally we show that α-synuclein A30P discriminating effectors are functional in full-length hemizygous and heterozygous models more closely resembling the disease setting indicating the suitability of the identified allele-specific sequences for future *in vivo* and potentially clinical work.

## Materials and Methods

### Constructs

All shRNA and target sequences can be found in supplementary data (Table S1). Oligonucleotides used to generate constructs were ordered from Sigma Genosys (Sigma Genosys, UK). All constructs were verified by sequencing prior to use.

### shRNA expression plasmids

shRNA expression plasmids were designed with antisense species located in the 3′ arm of stem-loop hairpins as described previously. To place shRNA sequences downstream of the U6 promoter, PCR was performed with Pol-III U6 promoter as template, a U6 forward primer (U6F': 5′-GATCGGGCCCGTCGACAAGGTCGGGCAGGAAGAGGGCCT-3′) and a U6 reverse primer containing shRNA sequences (U6R': 5′-AAAAAA.. Anti-sense ..TGGGTCAGG.. Sense ..GGTGTTTCGTCCTTTCCACAA-3′). PCR products were subsequently ligated into the pGEM-T Easy vector (Promega, USA) according to the manufacturer's instructions to generate RNA Pol-III expressed shRNA plasmids.

### siRNAs

All siRNAs were designed as 19-mers with 2-nt dT overhangs. The α-synuclein targeting siRNA p1314 was a kind gift from Novartis pharmaceuticals (Basel, Switzerland) with anti-sense sequence: 5′-UUGUCUUUCCUGGCGCUUCdTdT-3′. LRRK2 siRNAs p3-5 were from thermoscientific with the following antisense sequences: p3 5′-GCUGUAGUCAGCAAUCUUUdTdT-3′, p4 5′-UGCUGUAGUCAGCAAUCUUdTdT-3′, p5 5′-AUGCUGUAGUCAGCAAUCUdTdT-3′.

### Dual-luciferase targets

Complementary oligonucleotides containing partial length target sequences for dual-luciferase screening were annealed together and ligated into the multiple cloning site 3′ of the Renilla Luciferase gene in the psiCheck2.2 dual-luciferase cassette (Promega, USA) using Xho1 and Not1 restriction sites.

### Full-length α-synuclein constructs

Full-length wild-type and A30P mutant α-synuclein sequences cloned in the pcDNA-3.1 (Clontech, USA) plasmid were a kind gift from Dr J.Galvin (Washington, USA). Wild-type α-synuclein cloned in the peGFP-N1 plasmid was a kind gift from Dr A. Exposito (Cambridge, UK). The A30P mutant transcript was accordingly PCR amplified with forward and reverse primers containing SalI and SacII restriction sites respectively before being sub-cloned into the peGFP-N1 expression plasmid (Clontech, USA) using SalI and SacII restriction sites. To generate mCherry tagged constructs, mCherry was PCR amplified with forward and reverse primers containing SacII and NotI restriction sites respectively. Concomitantly, eGFP was removed by SacII and NotI digestion from α-synuclein eGFP-N1 plasmids. The mCherry PCR product was subsequently sub-cloned into α-synuclein plasmids with SacII and NotI restriction sites to generate α-synuclein mCherry-N1 expression plasmids. Finally, to generate heterozygous expression plasmids expressing eGFP-tagged wild-type α-synuclein and A30P mutant mCherry-tagged α-synuclein, mCherry-tagged A30P mutant α-synuclein together with a 5′ CMV promoter and 3′ poly-A tail were PCR amplified with forward and reverse primers containing PciI restriction sites. PCR products were subsequently sub-cloned into the wild-type α-synuclein peGFP-N1 plasmid using a single PciI restriction, and correct insert orientation verified by sequencing.

### Cell culture and transfections

HEK-293 cells (ATCC, CRL-1573) were cultured in DMEM supplemented with 10% FCS. For transfection, cells were grown in 24-well plates to 80% confluence and transfected with Lipofectamine 2000 (Invitrogen, USA) according to manufacturer's instruction. shRNAs and dual-luciferase targets were transfected at 1 µg/ml unless otherwise stated. For hemizygous assays, shRNAs and eGFP-tagged α-synuclein constructs were transfected at 1 µg/ml. For heterozygous assays shRNAs were transfected at 1 µg/ml, pri-miR-30 mimics at 0.5 µg/ml and the heterozygous expression plasmid at 2 µg/ml. For siRNA-1314 transfection, the heterozygous expression plasmid and siRNA-1314 were co-transfected at 2 µg/ml and 100 nM respectively. For LRRK2 siRNA transfections, 250 ng of psiCheck target plasmid was transfected with 50 nM of indicated siRNA unless stated otherwise.

### Luciferase assays

Cells were lysed for 20 minutes at time-points stated in the text using 100 µl passive lysis buffer (Promega, USA). A total of 20 µl of protein lysate was subsequently assayed for dual-luciferase readings using a dual-luciferase kit (Promega, USA) and Wallac-Victor 2 plate reader as per manufacturer's instructions. Ratios of renilla luciferase:firefly luciferase were obtained and normalised to respective non-specific control samples.

### Cell counting and viability assays

Cells were trypsinised 48 hrs post-transfection before cell number and typan blue determined cell viability were assessed with the Vi-Cell XR cell viability analyzer (Beckman Coulter) as per manufacturer's instructions.

### Rapid amplification of cDNA ends (RACE)

RNA was harvested at stated time-points using Trizol (Invitrogen, USA) according to manufacturers instructions. To define 5′ nucleotides of α-synuclein RNAi degradation products, 1 µM of RNA adapter with 5′ and 3′ hydroxyl groups (5′ OH-ACACUCUUUCCCUACACGACGCUCUUCCGAUCU-OH) was used in a ligation reaction with 250 ng of total RNA from α-synuclein mCherry-N1 and respective shRNA transfected HEK-293 cells. Following 1 hr reaction at 37°C with T4 RNA ligase (NEB, USA), 200 ng of RNA was reverse transcribed with thermoscript (Invitrogen, USA) and α-synuclein specific reverse primer (5′ CTGCTCCCTCCACTGTCTTC) according to the manufacturer's instruction. Resulting cDNA was diluted 1∶400 and 15 µl used as template in a 50 µl PCR reaction as previously described using adapter specific forward primer and nested α-synuclein specific reverse primer (F: 5′ AATGATACGGCGACCACCGAGATCTACACTCTTTCCCTACACGACGCTCTTCCGATCT, R: 5′ CCACTGCTCCTCCAACATTT). Products were visualised on a 2% agarose gel and cleavage products amplified prior to PCR clean-up and sequencing using band-stab PCR. RNAi cleavage sites were subsequently determined from sequencing reads by identifying junctions between adapter and α-synuclein transcripts.

### Fluorescent microscopy

Cells transfected with fluorescent constructs were visualised at stated time-points using a Leica DM-IRB inverted microscope (Leica, Germany). Images were taken with Carl Zeiss AxioCam MRm monochrome digital camera (Carl Zeiss, Germany) using AxioVision digital imaging software (Carl Zeiss, Germany). All images are 3600 µm in width.

### Fluorescent quantification

Cells transfected with fluorescent constructs were lysed at time points stated in the text and protein content determined using the micro BCA protein assay as per manufacturer's instructions (Pierce Biotechnology, USA). A volume of 100 µl was prepared in black-walled, clear-bottom 96-well plates (BD falcom) which contained 50 µg of protein lysate. Samples were subsequently assayed for eGFP and/or mCherry fluorescence using a fluorescent plate reader (Wallac Victor). Background fluorescence determined from cells transfected with target alone was subtracted. Fluorescence levels were subsequently normalised to non-specific control samples.

### Statistical analysis

Statistical significance between control and experimental values was determined using Student's *t* test (un-paired, 2-tailed). All data are expressed as mean ± standard deviation (S.D.).

## Results

### Identification of RNAi effectors for allele-specific targeting of α-synuclein A30P mutation

**Figure 1:**
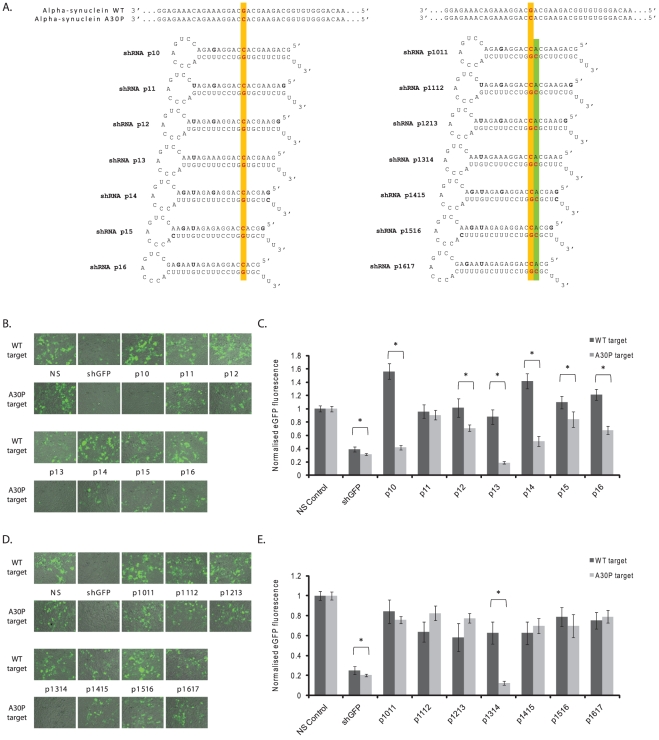
Screening of A30P-targeting shRNAs against full-length eGFP-tagged α-synuclein targets. A) shRNAs were designed targeting the A30P mutant allele of α-synuclein with the A30P mutation aligned at sequential positions in the 3′ region of the antisense species from positions 10–16 (P10-P16). In some shRNAs a secondary mismatch to the wild-type allele was additionally made immediately 3′ of the primary mismatch such that two mismatches are present to the wild-type target, and one mismatch to the A30P mutant target. B+D) Representative fluorescent images of HEK-293 cells co-transfected with stated eGFP-tagged α-synuclein targets and indicated single (B) or double (D) mismatch shRNA construct at 72 hrs post-transfection. C+E) Quantification of eGFP fluorescence at 72 hrs post-transfection following co-transfection of single (C) or double (E) mismatch shRNAs targeting the A30P α-synuclein mutant with wild-type (dark) or mutant (light) eGFP-tagged α-synuclein targets. Values represent mean ratios of normalized fluorescence +/− S.D. from n = 6. Values are normalized to cells transfected with non-specific shRNA and respective eGFP-tagged target. * = P<0.05 relative to respective normalising control.

Mutations to the α-synuclein encoding *SNCA* gene have been reported to cause early-onset PD in an autosomal dominant manner. To investigate the potential of RNAi as a therapeutic strategy for mutation carriers at the route of their disease, a panel of U6-transcribed shRNAs was designed which were fully complementary to the A30P mutant allele of α-synuclein and had a single G:G mismatch to the wild-type allele. Based on previous reports [8], [10], shRNAs were designed with the A30P mutation aligned at sequential positions along the 3′-region of the antisense strand from p10-16, with P1 representing the most 5̀nt of the antisense species (Fig. 1A). Some studies have demonstrated that discrimination can be improved when secondary mismatches to the wild-type allele are incorporated despite this leading to a single mismatch with the targeted mutant allele [6], [17]. To test this hypothesis, a second pool of shRNAs was designed in which a single U→C swap to create a single A:C mismatch to the mutant A30P allele, and a secondary mismatch to the wild-type allele, was placed immediately 3′ of the mutation alignment in the antisense strand (Fig. 1A).

Initially, full-length eGFP-tagged wild-type and A30P mutant α-synuclein constructs were designed and expressed in HEK-293 cells in hemizygous experiments. Western blot analysis confirmed expected sizes of the fusion proteins, and showed that the constructs represented a comprehensive overexpression of α-synuclein since no endogenous protein could be detected at the exposure times used (data not shown). Further, fluorescence microscopy confirmed robust expression whilst no intracellular aggregates of α-synuclein could be seen with either wild-type or mutant constructs at 72 hrs, agreeing with previously reported equivalent models [18]. Finally, cell counting and cell viability assays demonstrated no toxicity associated with these constructs relative to mock-transfected cells or relative to one another (Fig. S1).

To screen for allele-specific silencing, shRNAs were co-transfected with either wild-type or A30P mutant α-synuclein targets and eGFP expression was assessed and quantified at 48 hrs (Fig. S2) or 72 hrs (Fig. 1B, C). A positive control eGFP-targeting shRNA detectably reduced eGFP expression, and this was reproducibly quantified as a ∼60% reduction of both wild-type and mutant targets (Fig. 1C and S2). Importantly this demonstrates that both target constructs have the potential to be knocked down by non-mutation targeting shRNAs to almost identical levels, and implies that any difference seen in the silencing of the two different targets by A30P-targeted shRNAs is likely the result of the single-mismatch generated between the antisense species and the wild-type target. Analysis at both 48 and 72 hrs demonstrated that multiple constructs had the ability to silence one or both of the α-synuclein full-length targets (Fig. 1B and S2). In particular, constructs p10, p13 and p14 demonstrated striking discrimination when co-transfected with either the wild-type or A30P mutant constructs at both time points. The trends observed from eGFP-microscopy were verified by direct eGFP-quantification (Fig. 1C and S2). All constructs, with the exception of p11, displayed discrimination between mutant and wild-type constructs with the maximum difference being 4.65-fold (p<0.001) discrimination by construct p13 at 72 hrs. Further, each construct, with the exception of p11, displayed increased discrimination with time. p13 had the most substantial amount of mutant knockdown to 18%. Yet whilst p13 also showed silencing of the wild-type construct by 12%, both constructs p10 and p14 demonstrated high specificity with absent wild-type silencing accompanied by silencing of the mutant transcripts to 42% and 51% respectively.

Finally, screening of double-mismatch shRNAs was also carried out. The secondary mismatches both qualitatively (Fig. 1D and S2) and quantitatively improved the levels of wild-type retention relative to single mismatch shRNAs, but in nearly all cases this was accompanied by a greater retention of the mutant allele implying that the single mismatch to this sequence was not tolerated well by the RNAi machinery (Fig. 1E and S2). However, secondary-mismatch alignments to the wild-type allele at p1314 directed a marked reduction in the mutant protein to 20% and 12% of original levels at 48 and 72 hrs respectively, whilst the wild-type protein was completely retained at 48 hrs, but reduced to 60% at 72 hrs (Fig. 1E and S2). The resulting 5.8-fold (p<0.001) discrimination seen with this construct at 48 rs was the most impressive difference seen in this full-length assay and confirms that incorporation of an additional secondary mismatch can improve allele-specific discrimination of certain RNAi effectors.

### Allele-specific discrimination of the A30P mutant target in a heterozygous cell model

**Figure 2:**
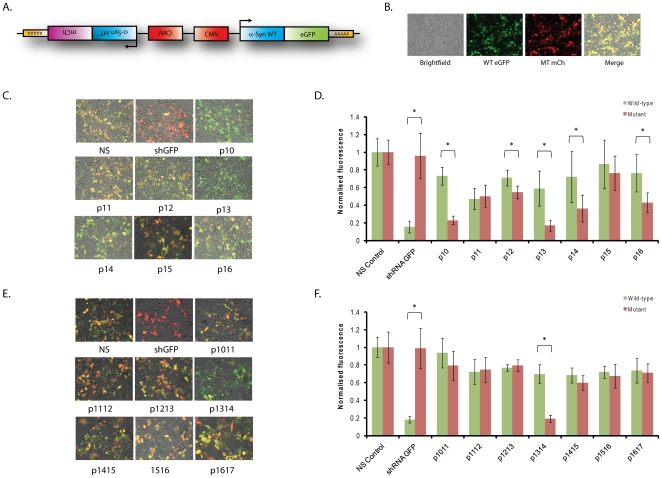
Screening of A30P-targeting shRNAs against the heterozygous eGFP-tagged wild-type and mCherry-tagged A30P mutant α-synuclein expression plasmid. A) eGFP-tagged wild-type α-synuclein and mCherry tagged A30P mutant α-synuclein were expressed in anti-parallel directions to one another in the Het A30P plasmid. B) Expression of the Het A30P plasmid resulted in robust eGFP fluorescence and robust mCherry fluorescence at 48 hrs post-transfection in HEK-293 cells. C+E) Representative merged fluorescent images of HEK-293 cells co-transfected with the Het-A30P plasmid and indicated single (C) or double (E) mismatch shRNA construct at 48 hrs post-transfection. D+F) Quantification of wild-type α-synuclein eGFP (green bars) or A30P mutant α-synuclein mCherry (red bars) fluorescence at 48 hrs post-transfection following co-transfection of single (D) or double (F) mismatch shRNAs targeting the A30P α-synuclein mutant with the Het-A30P plasmid. Values represent mean ratios of normalized fluorescence +/− S.D. from n = 6. Values are normalized to respective fluorescence in cells transfected with non-specific shRNA. * = P<0.05 relative to respective normalising control.

The autosomal dominant A30P mutation will result in both wild-type and mutant alleles being transcribed in patient cells. The silencing of either mRNA transcript will therefore be competitive under these conditions, and it is unknown how this will affect the allele-specific outcome. To investigate this a more complex model of α-synuclein overexpression was generated in which a eGFP-tagged wild-type and a mCherry-tagged A30P mutant transcript were expressed from the same plasmid using identical promoters located side-by-side, but directing anti-parallel transcription (Fig. 2A). This heterozygous A30P (HetA30P) arrangement was considered the most favourable for transcribing two constructs from one plasmid at equal levels with minimal interference of promoter activity on neighboring transcription units. Fluorescence microscopy at 48 hrs post-transfection revealed strong eGFP and mCherry fluorescence in HEK-293 cells to create a merged yellow pattern of expression (Fig. 2B), viability assays demonstrated that this plasmid presented no detectable toxicity relative to mock-transfections (Fig. S3) whilst qPCR confirmed that levels of both wild-type and mutant transcripts were comparable (data not shown).

Screening of all single and double mismatch shRNAs was performed against the HetA30P plasmid and both fluorescence microscopy and quantification for eGFP and mCherry were determined at 48 hrs post-transfection. An eGFP-targeting shRNA was used as a positive control for this system. This construct initiated a change from yellow to red colour in fluorescent images, reproducibly demonstrating ∼80% silencing of the eGFP-tagged wild-type construct whilst having no effect on the mCherry-tagged A30P mutant construct following quantification of fluorescence (Figs. 2C–F). The results highlight the sequence-specificity of RNAi and also the ability of this system to identify allele-specific silencing of the co-expressed target transcripts. Single mismatch shRNAs demonstrated that p10, p13 and p14 were once again the best discriminators between the wild-type and mutant alleles, with each construct leading to a predominant retention of green fluorescence in microscopy images (Fig. 2C). However, the discrimination was not as comprehensive as in hemizygous assays. Quantification of fluorescence revealed that mutant target expression was now reduced to 23%, 17% and 36% of control levels for p10, p13 and p14 respectively, but reductions in the wild-type transcript were now to 73%, 59% and 72% of control levels respectively (Fig. 2D). P13 still had the greatest discrimination of 3.4-fold (p<0.001) in this model, but this was not as substantial as the 4.3-fold discrimination at the same time point in hemizygous assays (Fig. S2). In recent years reports have suggested shRNAs may elicit toxic effects *in vivo* [19], [20]. To rule out the possibility that toxicity of individual shRNAs leading to cell death or cell cycle arrest was responsible for the observed changes in fluorescence, cell counting and viability assays were performed at the time of analysis on a variety of shRNAs including the most successful constructs, p10, p13 and p14. No significant differences were seen in cell number relative to mock-transfected cells, and only shRNA p10 showed a modest 12% decline in cell viability which is not expected to lead to significant changes in mutant fluorescence (Fig. S3).

Likewise, construct p1314 was the only double mismatch construct capable of allele-specific silencing using this model (Figs. 2E, F), but the 3.6-fold (p<0.001) discrimination between alleles was not as great as the 5.8-fold change seen at the same time point in the corresponding hemizygous assay due to the 30% reduction of the wild-type construct seen in this heterozygous model. As with single mismatch constructs, shRNA p1314 had no detectable effect on cell viability or cell number relative to mock-transfected cells (Fig. S3). Collectively the results demonstrate that RNAi effectors have been identified that discriminate impressively between wild-type α-synuclein and the A30P α-synuclein mutant in a heterozygous model which closely mimics the disease setting. Furthermore, the analysis of the silencing and discriminating ability of the most successful constructs across the two cellular models highlights the influence that competing wild-type and mutant transcripts may have on results, whilst additionally emphasising that constructs p13 and p1314 are the most consistent discriminators and potentially suitable for future *in vivo* and clinical application.

### siRNA analysis and 5′RACE of target degradation products confirms the alignments of the mutation in antisense species

In order to put the findings with respect to allele-specific silencing of mutant A30P α-synuclein into perspective it is important that the precise alignment of mutations in the antisense strand is verified. Unlike siRNAs, shRNAs are subject to processing by Dicer to produce the dsRNA duplex from which the active antisense species is selected. Whilst the shRNAs were designed to release a sequence with the mutation alignment at the stated position based on known criteria for Dicer recognition, this remains to be verified. Here, two different approaches were utilised to verify the sequence alignments of mutations in the generated antisense species.

**Figure 3:**
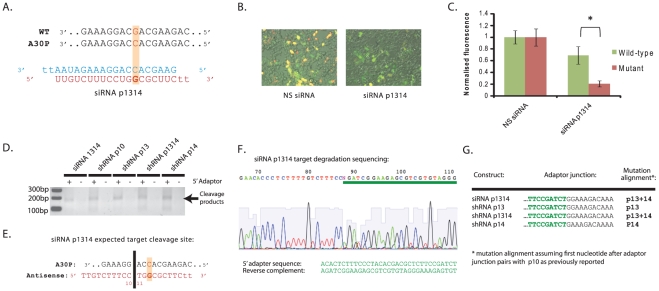
Determination of A30P mutation alignment with siRNA and 5′RACE. A) siRNA design with A30P mutation aligned opposite P13 of the antisense species, and secondary mismatch to the wild-type α-synuclein allele at P14. B) Representative merged fluorescent images of HEK-293 cells co-transfected with the Het-A30P plasmid siRNA-1314 at 48 hrs post-transfection. C) Quantification of wild-type α-synuclein eGFP (green bars) or A30P mutant α-synuclein mCherry (red bars) fluorescence at 48 hrs post-transfection following co-transfection of siRNA-1314 with the Het-A30P plasmid. Values represent mean ratios of normalized fluorescence +/− S.D. from n = 6. Values are normalized to respective fluorescence in cells transfected with non-specific siRNA. * = P<0.05 relative to respective normalising control. D) Visualisation of PCR products following 5′RACE using RNA from cells transfected with mCherry-tagged A30P mutant α-synuclein and stated constructs. Products were run on a 2% agarose gel. E) Expected target cleavage site for siRNA-1314. F) Sequencing of siRNA-1314 PCR product following 5′RACE. G) Mapping of 5′ adaptor ligations sites, determination of target cleavage sites and determination of A30P mutation alignments in stated constructs.

First, a siRNA bearing a primary mutation at p13 of the antisense species, and secondary mismatch at p14 was tested for allele-specific silencing ability to compare activity with the previously identified shRNA construct p1314 (Fig. 3A). The double mismatch siRNA-p1314 was chosen over a single mismatch variant since it was clear from results in the previous models that only one double mismatch construct was capable of allele-specific silencing whilst all other double mismatch variants failed to silence the wild-type and mutant transcripts. Thus, if siRNA-p1314 was capable of allele-specific discrimination then it is highly likely that shRNA p1314 has the primary mutation aligned correctly at p13 whilst other constructs have the mutation aligned accordingly. Transfection of siRNA-p1314 with the HetA30P plasmid demonstrated that the mutant A30P target was silenced by 79% and the wild-type target by 31% to produce a 3.4-fold (p<0.001) allele-specific discrimination (Figs. 3B, C). These results correlate closely with the 81% silencing of the mutant target and 30% reduction in wild-type target observed with shRNA p1314 at 48 hrs which strongly suggests that the alignments of mutations in the shRNAs are as predicted.

Rapid amplification of cDNA ends (RACE) has previously been used to determine the precise cleavage site of antisense species by determining the 5′ terminal nt of 3′ target cleavage products. Assuming cleavage of fully complementary targets is always directed opposite from p10 and p11 as reported [21] (Fig. 3E), comparison of alignments to shRNA sequences can subsequently be used to confirm the cleavage site of Dicer within the shRNA and hence the alignments of specific nucleotides in the antisense species. A 5′RACE protocol was used in which an RNA adapter was ligated to 3′ degradation products of the mCherry-tagged A30P mutant construct following co-transfection of constructs siRNA-p1314, p13, p1314 and p14, and then RT-PCR carried out with adaptor and α-synuclein specific primers. This adapter ligation exploits unique RNA ends and is only expected to ligate to RNA populations with 5′ phosphate groups. This includes RNAi degradation products but not full-length mRNAs which have 7-methylguanosine caps at the 5′ end.

Following 5′ RACE, gel-electrophoresis revealed that a band of the expected size for degradation products, but not the full-length transcript, was seen with all constructs (Fig. 3D). Importantly this was absent if the RNA-adaptor was excluded from ligation reactions. Sequencing of PCR products revealed first that the product was indeed that of α-synuclein degradation products, and second that the 5̀nt of the degradation products varied between effectors with the primary mutation aligned at P13 and P14 to confirm that the cleavage site within α-synuclein changes between constructs (Figs. 3F, G). More specifically the 5̀nt of the cleavage product aligned to the 11^th^ nt of each specific antisense species, including with siRNA-p1314 with which the alignment is verified. This demonstrates that cleavage is occurring opposite nucleotides p10 and p11 for each construct, and that the sequence alignments are as predicted.

### Dual-luciferase screening reveals shRNAs that can discriminate the LRRK2 G2019S mutant allele from the wild-type allele

**Figure 4:**
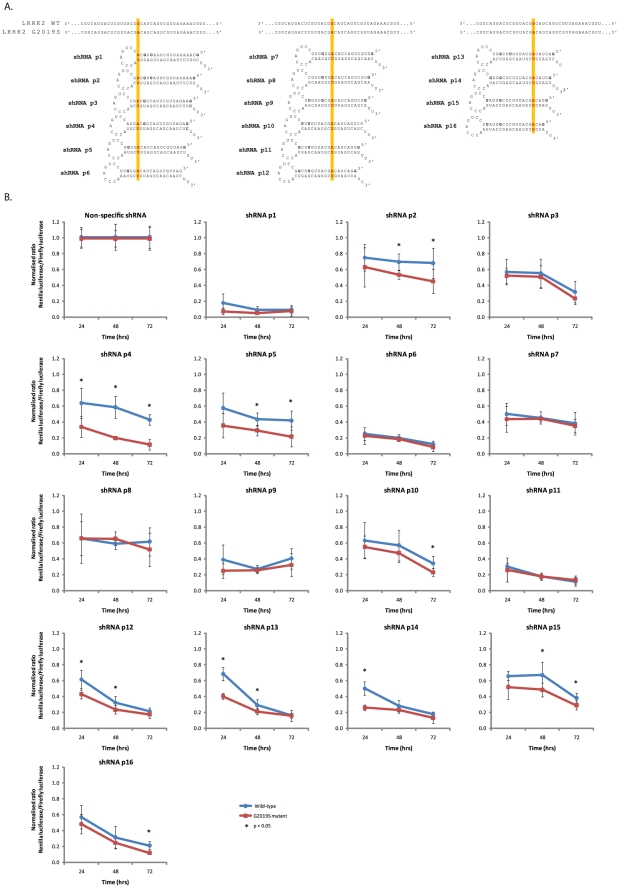
Screening of LRRK2 G2019S-targeting shRNAs against dual-luciferase targets. A) shRNAs were designed targeting the G2019S mutant allele of LRRK2 with the G2019S mutation aligned at sequential positions from position 1–16 (P1–P16) of the antisense species. B) Dual-luciferase assays at indicated time-points with stated shRNAs targeting the G2019S LRRK2 mutant following co-transfection with wild-type (blue lines) or mutant (red lines) luciferase targets. Values represent mean ratios of *Renilla*:Firefly luciferase +/− S.D. from n = 6. Values are normalized to cells transfected with non-specific shRNA and respective luciferase target. * = P<0.05 relative to respective normalising control.

Following the successful allele-specific discrimination achieved with shRNAs targeting the A30P α-synuclein mutation, a second PD candidate mutation was chosen for shRNA screening. The LRRK2 G2019S mutation is the most common PD-linked mutation currently known and therefore represents the most attractive mutation for allele-specific silencing. This mutation leads to a G:A conversion in the LRRK2 mRNA which results in a G:U mismatch between the antisense species of targeting RNAi effectors and the wild-type allele. A pool of shRNAs was initially designed which was fully complementary to the G2019S mutant allele of LRRK2 resulting in a single G:U mismatch to the wild-type allele mutation aligned at sequential positions from p10-16 of the antisense arm (Fig. 4A). However, kinetic studies on RNAi suggest that alignments in the 5′ region of the antisense species could lead to improved discrimination of the G2019S mutation. Specifically, G:U wobbles between the targeting shRNA and wild-type allele, have been reported to strongly interfere with pairings of antisense species to mRNA when placed either 5′ or centrally in the RNAi effector sequence [22], [23]. Accordingly, shRNAs were subsequently designed with mutations at sequential positions from P1-9 of the antisense arm.

There remains an acknowledged paucity of experimental models of LRRK2 that have made its study troublesome to date [24]. In order to rapidly screen shRNAs for allele-specific silencing ability, short wild-type and mutant allele target sequences corresponding to the 52nts of LRRK2 mRNA immediately surrounding the location of the G2019S mutation were therefore inserted into the 3̀UTR of the Renilla luciferase gene in a dual-luciferase vector. Screening of shRNAs against partial-length LRRK2 dual-luciferase targets revealed that at 24 hrs post-transfection, four shRNAs displayed significant allele-specific discrimination (Fig. 4B). Alignments of the G2019S mutation at p4, p12, p13 and p14 displayed 1.88- (p<0.005), 1.44- (p<0.005), 1.70- (p<0.001) and 1.93-fold (p<0.001) discrimination respectively, with p14 producing the maximum levels of mutant silencing of these three constructs to 26% whilst retaining 50% of the wild-type allele expression. Analysis at different points post-transfection revealed that all shRNAs led to increased levels of silencing of both the mutant and wild-type alleles over time (Fig. 4B). At 72 hrs, six shRNAs displayed significant discrimination between the mutant and wild-type alleles; p2, p4, p5, p10, p15 and p16. Construct p4 displayed the greatest discrimination (3.7-fold; p<0.001) between mutant and wild-type alleles, and was the only construct which maintained discrimination at all time points. Further, this shRNA p4 had no effect on cell number or cell viability relative to mock-transfected cells to rule out changes in luminescence being dependent on non-specific effects of cell death or cell cycle arrest (Fig. S3).

**Figure 5:**
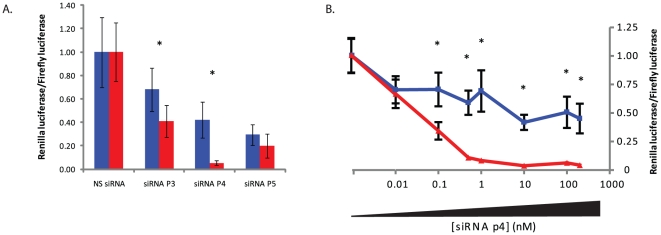
Screening of LRRK2 G2019S-targeting siRNAs against dual-luciferase targets. A) Dual-luciferase assays at 48 hrs with stated siRNAs targeting the G2019S LRRK2 mutant following co-transfection with wild-type (blue lines) or mutant (red lines) luciferase targets. B) Dual-luciferase assays at 48 hrs with siRNA p4 targeting the G2019S LRRK2 mutant at stated concentration following co-transfection with wild-type (blue lines) or mutant (red lines) luciferase targets. Values represent mean ratios of *Renilla*:Firefly luciferase +/− S.D. from n = 6. Values are normalized to cells transfected with non-specific shRNA and respective luciferase target. * = P<0.05 relative to respective normalising control.

In order to verify the sequence alignment of the G2019S mutation in the generated antisense species of the p4 construct, siRNAs with alignment of the mutations at p3, p4 and p5 were screened against the luciferase targets. At 48 hrs post-transfection siRNA p4 displayed a 7.7-fold (p<0.001) discrimination that was improved upon that seen with shRNA p4 at this time point (Fig 5A). In contrast siRNAs p3 and p5 displayed limited, if any, discrimination between the two alleles, again agreeing with the trends from previous shRNA data which showed alignment at p4 to be superior to these two constructs. Further, discrimination by siRNA p4 was evident using siRNA concentrations as low as 0.1 nM, and was increased to >8-fold by concentrations of siRNA greater than 1 nM in separate experiments (Fig 5B). The greatest discrimination was 10.8-fold (p<0.001) when using 10 nM siRNA, and at this concentration a 96% silencing of the mutant target was seen which was accompanied by a modest 58% silencing of the wild-type target. However, the greatest difference in target silencing was the 61% difference between the 92% silencing of the mutant target and 31% silencing of the wild-type target when using 1 nM siRNA. Collectively this data strongly suggests that the alignment of the G2019S mutation in shRNA p4 was as stated, whilst additionally demonstrating that siRNA p4 has impressive and potent discriminating ability that could be useful in future pre-clinical models of G2019S associated pathology.

### Secondary mismatches at nucleotide positions opposite the target cleavage site can improve the discrimination of G2019S targeting shRNAs

**Figure 6:**
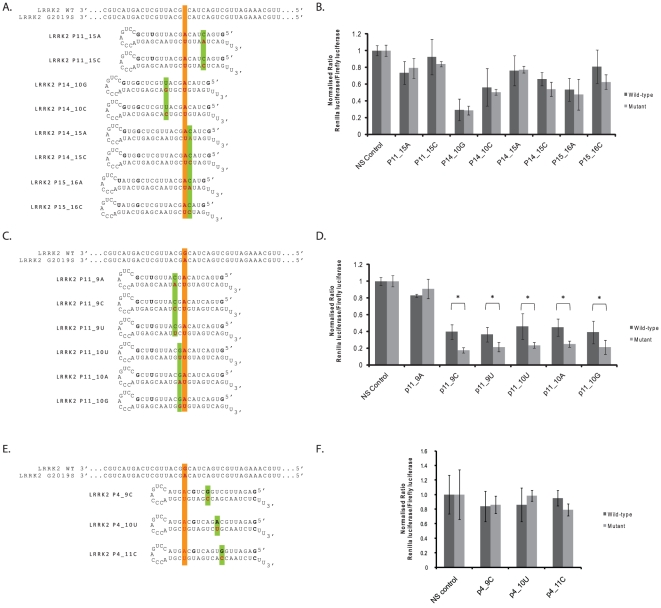
Screening of G2019S-targeting shRNAs incorporating secondary mismatches to wild-type LRRK2 against dual-luciferase targets. A, C and E) shRNAs were designed targeting the G2019S mutant allele of LRRK2 with the G2019S mutation aligned at stated positions in the 3′ region of the antisense species. Secondary mismatches to the wild-type allele were additionally made at indicated positions in the antisense species such that two mismatches are present to the wild-type target, and one mismatch to the G2019S mutant target. B, D and F) Dual-luciferase reporter assays at 48 hrs post-transfection with double mismatch shRNAs targeting the G2019S LRRK2 mutant following co-transfection with wild-type (dark bars) or mutant (light bars) luciferase targets. Values represent mean ratios of *Renilla*:Firefly luciferase +/− S.D. from n = 6. Values are normalized to cells transfected with non-specific shRNA and respective luciferase target. * = P<0.05 relative to respective normalising control.

Despite allele-discrimination being evident with single-mismatch shRNAs targeting the G2019S mutation, particularly at p4, substantial silencing of the wild-type allele is also observed. Following the previous demonstration that secondary mismatches could improve the discrimination between mutant and wild-type targets in this and other reports [6], [17], a series of shRNAs containing one mismatch to the G2019S mutant allele and a secondary mismatch to the wild-type allele was initially designed to attempt to improve allele-specific discrimination of those constructs aligned in 3′ regions of the antisense species (Figs. 6A, C).

Initially a series of constructs was designed with the secondary mismatch placed directly 5′ or 3′ of the G2019S mutation alignment; p11_10, p14_15, p15_16 (Figs. 6A, C). The primary mutation alignments chosen in these constructs corresponded to those single-mismatch constructs with which greatest knockdown of the mutant allele was consistently seen (p11 and p14) in single mismatch assays, or with which the least silencing of the wild-type target was seen (p15). In addition to these constructs others were designed in which the most effective single mismatch constructs were paired with a separated secondary mismatch placed at alignments which showed limited silencing of the wild-type targets (p11_15, p14_10) (Fig. 6A). Further, the shRNA with primary alignment at p11, consistently one of the constructs which silenced the mutant allele to the greatest extent, was paired with secondary mismatches at p9 or p10 directly opposite where target cleavage is predicted to occur (Fig. 6C). This was under the hypothesis that this may interfere with target cleavage directly by producing an unstable platform around the cleavage site. Finally, all constructs were designed both with either hypothetically “weak” or hypothetically “strong” secondary mismatches to the target based on previously published data [8] (Figs. 6A, C), whilst secondary mismatches around the target cleavage site were designed in every possible variation of mismatch at these positions (Fig. 6C).

Analysis of silencing at 48 hrs post-transfection demonstrates that secondary mismatches made to shRNAs p11, p14, and p15 which were not surrounding the target cleavage site failed to improve allele-specific discrimination (Fig. 6B). As expected the silencing ability of the wild-type target was reduced with these constructs, but the mutant target also demonstrated reduced silencing due to the single mismatch to its sequence. An additional observation was that, aside from variants of construct p1415, a trend was seen that the theoretically stronger secondary mismatches for allele-specific silencing were able to impair the silencing of both targets to the greatest extent (Fig. 6B). Encouragingly, the secondary mismatches introduced into the p11 construct that were located at p9 or p10 were able to improve discrimination (Fig. 6D). Apart from construct p11_9A, which showed a heavily reduced ability to silence both wild-type and mutant targets, all other constructs with secondary mismatches introduced at p9 or p10 maintained the ability to silence the mutant target by ∼80%. This was coupled to a significantly reduced ability to silence the wild-type target leading to highly significant 2.23- (p<0.001), 1.71- (p = 0.02), 1.96- (p = 0.01), 1.81- (p = 0.009) and 1.83-fold (p = 0.01) discrimination for constructs p11_9C, p11_9U, p11_10U, p11_10A, p11_10G respectively. Whilst this is a marked improvement on the ability to discriminate by the p11 construct, a marked knockdown of the wild-type construct was seen in all these cases by at least 54% or more. These levels of discrimination were not as substantial as the 2.94-fold (p<0.001) discrimination at this time point that was achieved with single mismatch shRNA construct p4, but do provide new secondary mismatch design guidelines. Accordingly three variants of shRNA p4 were made in which a secondary mismatch was placed at either p9, p10 or p11 (Fig. 6E). In this instance only the weakest possible mismatch based on previously published data was used for each construct to try and minimise loss of silencing of the mutant transcript. However, in all cases the secondary mismatch acted to decrease silencing of both the wild-type and mutant targets, with no improvement in discrimination seen (Fig. 6F).

## Discussion

The recent identification of several genes with genetic linkage to PD provides valuable insight into the underlying aetiology of the disease [1]. In addition, this knowledge has revealed several novel therapeutic targets for PD, both for familial and sporadic forms of the disease, and the unique chance to target the direct cause of the disease in certain patients. Here we made the first steps towards investigating allele-specific silencing using RNAi as a novel therapy with the potential to achieve this goal of personalized medicine for certain PD patients in future. The results demonstrate that allele-specific RNAi is possible for the α-synuclein A30P and LRRK2 G2019S PD-linked mutations, with alignments p10, p13, p14 and p1314 best for the A30P mutation and p4 best for the G2019S mutation, but that the success of this approach is highly dependent on the nature of the mutation. However, it is demonstrated that discriminating ability of allele-specific RNAi effectors may be improved with incorporation of secondary mismatches, findings that will be applicable to this field of RNAi research in future.

The A30P α-synuclein mutation is a rare PD-associated mutation, and increased rates of formation of α-synuclein fibrils and/or intermediate toxic proto-fibrils in addition to other pathogenic mechanisms resulting from this mutation suggest that silencing would be beneficial patients with this variant [25]–[27]. Non-allele specific α-synuclein RNAi has been investigated as a potential approach for PD through *in vivo* delivery of shRNAs to the rat substantia nigra pars compacta [28]. However this indiscriminate silencing was associated with nigrostriatal degeneration which agrees with findings from transgenic α-synuclein null mice showing disturbances to the nigrostriatal system [29]. Thus whilst silencing of α-synuclein is expected to ameliorate α-synuclein pathology it may enhance the dopaminergic deficit responsible for the characteristic motor phenotypes of PD patients. Targeted reduction of the mutant α-synuclein transcripts should therefore be considered the ideal strategy for treating these hereditary forms of PD bearing mutations in α-synuclein due to sparing of the essential wild-type function.

Several shRNA constructs with mutations aligned across 3′ regions of the antisense species were capable of silencing the α-synuclein A30P mutation whilst sparing the wild-type allele in both models tested, including a heterozygous model designed to replicate the disease setting in which both wild-type and mutant alleles are present [14]. Success with 3′ alignments is consistent with previous studies showing good discrimination at such positions [8], [10], although good discrimination was seen with several alignments and not just the strong preference for alignment at p16 suggested by Schwarz et al. [8]. Whilst phenotypic models of A30P pathology [30], [31] could not be replicated in our hands, it is likely that the strong discrimination seen against the A30P mutation with some constructs would be therapeutically beneficial. It will be interesting to test these constructs in transgenic mouse models carrying this mutation in future to see if phenotypic correction can be achieved [32], [33].

Estimates for the incidence of the LRRK2 G2019S mutation are presently ∼1% of all PD patients making it an attractive mutation to target [1], [16]. The mutation leads to a gain-in-function of the kinase domain to suggest that reducing kinase activity would be therapeutically beneficial [34]. Despite transgenic null mice displaying no gross central nervous system abnormalities [35], at present little is known about the function of LRRK2 whilst GWAS studies imply abnormalities at the LRRK2 locus are a cause of non-hereditary PD to stress its potential importance in disease pathology [2], [3]. As such allele specific silencing would be considered preferable over non-allele specific silencing due to LRRK2′s presently unknown activity, and this is the first report where this mutation has been successfully targeted. A recent publication of allele-specific discrimination of this mutation with siRNAs has been made. However, the G2019S model initially reported online contains a 6056G→A transition rather than the 6055G→A transition originally reported in the LRRK2 coding sequence [36], and siRNAs appear designed based on this incorrect mutation [37]. A mistake in manuscript preparation has been confirmed, and this likely explains why discrimination was reported at P10, P11, P14 and P16 which each failed to show discrimination in this study.

In this study discrimination was seen between wild-type and mutant alleles in hemizygous dual-luciferase assays, but the differences observed were relatively modest and reduced with time. The exception to this was when the mutation was aligned at p4 of the antisense strand. Discrimination of 2.94- and 3.7-fold were seen at 48 and 72 hrs respectively with shRNA p4, and greater than 10-fold was observed when using siRNA p4 at 48 hrs. It remains to be seen if the discrimination levels observed would have therapeutic benefit. One concern with the dual-luciferase system is the absence of similar secondary structure to the desired targets which could have an effect on silencing ability [38], [39]. It is possible that in this study the secondary structure surrounding the G2019S target sequence is particularly relaxed and accessible to RNAi. Whether this is the same with the endogenous full-length LRRK2 sequence surrounding the G2019S mutation is unclear. However, influence of secondary structure on RNAi is still debated since the formation of the A-form helix recognised by the RNA induced silencing complex (RISC) dictates that any secondary structure would have to be released prior to RNAi [40], [41]. It will therefore be imperative to test this p4 construct and future secondary mismatch variants displaying good discrimination in more advanced models such as primary patient cells or the recently reported G2019S transgenic mouse [42].

Reports have been published of shRNAs leading to significant toxicity *in vivo* which can have fatal consequences in some animal models [19], [20]. Whilst no toxicity was associated with shRNA p4, or indeed any of the shRNAs tested in this study, the incorporation of antisense species into pri-miRNA backbones has been highlighted as the safest expressed RNAi effector presently available due to an apparent regulatory role of the Drosha/DGCR8 microprocessor in limiting accumulation of toxic pre-miRNA species [20], [43]. Whilst shRNAs targeting the A30P mutation may not merit translation into pri-miRNA mimics, the incidence of the LRRK2 G2019S mutation suggests that development of the p4 antisense sequence into pri-miRNA mimics should be strongly considered in future studies.

The success in targeting the α-synuclein A30P mutation is unsurprising given the resulting G:G mismatch that is present between the wild-type allele and antisense species, a mismatch that has been demonstrated previously to be one of the most favourable for allele-specific silencing purposes [6], [8], [9]. It is expected that the purine:purine alignment strongly disrupts the A-form helix that is usually formed following pairing of the antisense species to the target due to the presence of two dual-ring nitrogenous bases aligned between the sugar-phosphate backbones where usually a dual-ringed purine and a single-ringed pyrimidine exist.

It is expected that the reason for limited discrimination between alleles when targeting the LRRK2 G2019S mutation with most alignments was the result of a weaker mismatch being present between the antisense species and the wild-type allele. The resulting G:U alignment is commonly encountered within RNA biology [44], and has thermodynamic stability approaching that of normal Watson–Crick base pairs which exceeds almost all other mismatches [45]. Indeed previous rules determined for allele-specific silencing found the G:U wobble to be the second weakest alignment for discrimination that wasn't Watson-Crick base-pairing [8], although others have demonstrated allele-specific silencing of G:U mismatches [6], [7], [10], [12]. However, alignment at p4 in the 5′ region of the antisense species showed improved discrimination of the G2019S mutation when using both shRNAs and siRNAs. This fits with previous reports indicating introduction of G:U wobbles strongly interferes with pairings of antisense species to mRNA when placed either 5′ or centrally in the RNAi effector [22], [23], although this is not a consensus view at present [46].

Finally, it was confirmed that secondary mismatches could improve allele-specific discrimination of RNAi effectors. Given the improved discrimination by the α-synuclein targeting construct P1314 over the single mismatch construct P13, it was disappointing not to see a more marked improvement with the tested secondary mismatches in shRNAs targeting the G2019S mutation. No definitive rules have previously been established for incorporation of secondary mismatches to enhance allele-specific discrimination despite previous success with this approach [6], [17], and attempts were made here to determine these by testing multiple design strategies against the G2019S mutation. Placing a secondary mismatch opposite the target cleavage site improved discrimination relative to the single mismatch constructs in some shRNAs, but not all, and this trend supports early allele-specific studies which hypothesised that alignment of mutations at such positions was likely to have the most disruptive effect on RNAi activity [6], [7], [9], [12]. Importantly it also demonstrates that extensive screening of variants may often be needed before suitable variants are found. Finally, in the successful examples, the strongest effect was seen when this secondary position was changed from a G:C alignment to a C:C mismatch, and this agrees with this alignment being more disruptive than both a A:C mismatch and a U:C mismatch in previous studies [8]. This suggests that if secondary mismatches are to be used in future, then the “strength” of the secondary mismatch should be considered as this can influence the success.

In summary we have identified several RNAi constructs with the ability to discriminate wild-type and mutant alleles of two PD-linked genes. The results agree with previous allele-specific silencing rules suggesting that the nature of the mutations has a strong influence on the success of this approach, whilst we confirm that secondary mismatches can be used to improve discrimination further in some cases. It remains to be seen whether the constructs identified are therapeutically beneficial, and the identified constructs now await evaluation and development in more advanced pre-clinical models to test for therapeutic efficacy.

## Supporting Information

Figure S1 **Cell counting and cell viability from cells expressing eGFP-tagged α-synuclein targets.** A) Cell counts from HEK-293 cells transfected with stated eGFP-tagged α-synuclein targets or mock transfection at 48 hrs post-transfection. B) Trypan blue cell viability assay of HEK-293 cells transfected with stated eGFP-tagged α-synuclein targets or mock transfection at 48 hrs post-transfection.
(EPS)

Figure S2 **Screening of A30P-targeting shRNAs against full-length eGFP-tagged α-synuclein targets.** A+C) Representative fluorescent images of HEK-293 cells co-transfected with stated eGFP-tagged α-synuclein targets and indicated single (A) or double (C) mismatch shRNA construct at 48 hrs post-transfection. B+D) Quantification of eGFP fluorescence at 48 hrs post-transfection following co-transfection of single (B) or double (D) mismatch shRNAs targeting the A30P α-synuclein mutant with wild-type (dark bars) or mutant (light bars) eGFP-tagged α-synuclein targets. Values represent mean ratios of normalized fluorescence +/− S.D. from n = 6. Values are normalized to cells transfected with non-specific shRNA and respective eGFP-tagged target. * = P<0.05 relative to respective normalising control.
(EPS)

Figure S3 **Cell counting and cell viability from cells expressing shRNAs and corresponding targets.** A) Cell counts from mock transfected HEK-293 cells or HEK-293 cells transfected with stated het-A30P plasmid and indicated shRNAs at 48 hrs post-transfection. B) Trypan blue cell viability assay of mock transfected HEK-293 cells or HEK-293 cells transfected with stated het-A30P plasmid and indicated shRNAs at 48 hrs post-transfection. C) Cell counts from mock transfected HEK-293 cells or HEK-293 cells transfected with G2019S dual-luciferase target plasmid and indicated shRNAs at 48 hrs post-transfection. D) Trypan blue cell viability assay of mock transfected HEK-293 cells or HEK-293 cells transfected with G2019S dual-luciferase target plasmid and indicated shRNAs at 48 hrs post-transfection.
(EPS)

Table S1 **Oligonucleotide sequences used.**
(DOC)
