# Supplementary material for: Identification of Allele-Specific RNAi Effectors Targeting Genetic Forms of Parkinson's Disease
Source: PLoS One. 2011 Oct 21;6(10):e26194. doi: 10.1371/journal.pone.0026194 (PMC3198729; doi:10.1371/journal.pone.0026194)
Supplement: Table S1 — Oligonucleotide sequences used. (DOC) [file pone.0026194.s004.doc]

| **Construct:** | **Sequence:** |
| --- | --- |
|  | **shRNA R’ primers** |
| α-SYN P10 | 5’ AAAAAAGCAGAAGCACCAGGAAAGATGGGTCAGGTCTCTCCTGGTGCTTCTGCGGTGTTTCGTCCTTTCCACAA |
| α-SYN P11 | 5’ AAAAAACAGAAGCACCAGGAAAGACTGGGTCAGGATCTCTCCTGGTGCTTCTCGGTGTTTCGTCCTTTCCACAA |
| α-SYN P12 | 5’ AAAAAAAGAAGCACCAGGAAAGACATGGGTCAGGTATCTCTCCTGGTGCTTCCGGTGTTTCGTCCTTTCCACAA |
| α-SYN P13 | 5’ AAAAAAGAAGCACCAGGAAAGACAATGGGTCAGGTTATCTTTCCTGGTGCTTCGGTGTTTCGTCCTTTCCACAA |
| α-SYN P14 | 5’ AAAAAAGAGCACCAGGAAAGACAAATGGGTCAGGTCTATCTTTCCTGGTGCTCGGTGTTTCGTCCTTTCCACAA |
| α-SYN P15 | 5’ AAAAAAAGCACCAGGAAAGACAAAGTGGGTCAGGTTCTATCTTTCCTGGTGCCGGTGTTTCGTCCTTTCCACAA |
| α-SYN P16 | 5’ AAAAAAGCACCAGGAAAGACAAAAGTGGGTCAGGCTCTTATCTTTCCTGGTGCGGTGTTTCGTCCTTTCCACAA |
| α-SYN P1011 | 5’ AAAAAAGCAGAAGCGCCAGGAAAGATGGGTCAGGTCTCTCCTGGTGCTTCTGCGGTGTTTCGTCCTTTCCACAA |
| α-SYN P1112 | 5’ AAAAAACAGAAGCGCCAGGAAAGACTGGGTCAGGATCTCTCCTGGTGCTTCTCGGTGTTTCGTCCTTTCCACAA |
| α-SYN P1213 | 5’ AAAAAAAGAAGCGCCAGGAAAGACATGGGTCAGGTATCTCTCCTGGTGCTTCCGGTGTTTCGTCCTTTCCACAA |
| α-SYN P1314 | 5’ AAAAAAGAAGCGCCAGGAAAGACAATGGGTCAGGTTATCTTTCCTGGTGCTTCGGTGTTTCGTCCTTTCCACAA |
| α-SYN P1415 | 5’ AAAAAAGAGCGCCAGGAAAGACAAATGGGTCAGGTCTATCTTTCCTGGTGCTCGGTGTTTCGTCCTTTCCACAA |
| α-SYN P1516 | 5’ AAAAAAAGCGCCAGGAAAGACAAAGTGGGTCAGGTTCTATCTTTCCTGGTGCCGGTGTTTCGTCCTTTCCACAA |
| α-SYN P1617 | 5’ AAAAAAGCGCCAGGAAAGACAAAAGTGGGTCAGGCTCTTATCTTTCCTGGTGCGGTGTTTCGTCCTTTCCACAA |
| LRRK2 P1 | 5’ AAAAAAGCAAAGATTGCTGACTACATGGGTCAGGTGCAATCAGCAATCTTTGCGGTGTTTCGTCCTTTCCACAA |
| LRRK2 P2 | 5’ AAAAAACAAAGATTGCTGACTACAGTGGGTCAGGCTGCAATCAGCAATCTTTCGGTGTTTCGTCCTTTCCACAA |
| LRRK2 P3 | 5’ AAAAAAGAAGATTGCTGACTACAGCTGGGTCAGGGCTGCAGTCAGCAATCTTCGGTGTTTCGTCCTTTCCACAA |
| LRRK2 P4 | 5’ AAAAAAGAGATTGCTGACTACAGCATGGGTCAGGTACTGCAGTCAGCAATCTCGGTGTTTCGTCCTTTCCACAA |
| LRRK2 P5 | 5’ AAAAAAAGATTGCTGACTACAGCATTGGGTCAGGACACTGTAGTCAGCAATCCGGTGTTTCGTCCTTTCCACAA |
| LRRK2 P6 | 5’ AAAAAAGATTGCTGACTACAGCATTTGGGTCAGGAACACTGTAGTCAGCAATCGGTGTTTCGTCCTTTCCACAA |
| LRRK2 P7 | 5’ AAAAAAATTGCTGACTACAGCATTGTGGGTCAGGCAACACTGTAGTCAGCAACGGTGTTTCGTCCTTTCCACAA |
| LRRK2 P8 | 5; AAAAAATTGCTGACTACAGCATTGCTGGGTCAGGGCAACACTGTAGTCAGCACGGTGTTTCGTCCTTTCCACAA |
| LRRK2 P9 | 5’ AAAAAATGCTGACTACAGCATTGCTTGGGTCAGGAACAACGCTGTAGTCAGCCGGTGTTTCGTCCTTTCCACAA |
| LRRK2 P10 | 5’ AAAAAAGCTGACTACAGCATTGCTCTGGGTCAGGAAACAATGCTGTAGTCAGCGGTGTTTCGTCCTTTCCACAA |
| LRRK2 P11 | 5’ AAAAAACTGACTACAGCATTGCTCATGGGTCAGGCGAACAATGCTGTAGTCACGGTGTTTCGTCCTTTCCACAA |
| LRRK2 P12 | 5’ AAAAAATGACTACAGCATTGCTCAGTGGGTCAGGCCGAACAATGCTGTAGTCCGGTGTTTCGTCCTTTCCACAA |
| LRRK2 P13 | 5’ AAAAAAGACTACAGCATTGCTCAGTTGGGTCAGGACCGAACAATGCTGTAGTCGGTGTTTCGTCCTTTCCACAA |
| LRRK2 P14 | 5’ AAAAAAACTACAGCATTGCTCAGTATGGGTCAGGCACCGAGCAATGCTGTAGCGGTGTTTCGTCCTTTCCACAA |
| LRRK2 P15 | 5’ AAAAAACTACAGCATTGCTCAGTACTGGGTCAGGATACCGAGCAATGCTGTACGGTGTTTCGTCCTTTCCACAA |
| LRRK2 P16 | 5’ AAAAAATACAGCATTGCTCAGTACTTGGGTCAGGAATACCGAGCAATGCTGTCGGTGTTTCGTCCTTTCCACAA |
| LRRK2 P11_15A | 5’ AAAAAACTGATTACAGCATTGCTCATGGGTCAGGCGAACAATGCTGTAGTCACGGTGTTTCGTCCTTTCCACAA |
| LRRK2 P11_15C | 5’ AAAAAACTGAATACAGCATTGCTCATGGGTCAGGCGAACAATGCTGTAGTCACGGTGTTTCGTCCTTTCCACAA |
| LRRK2 P14_10G | 5’ AAAAAAACTACAGCACTGCTCAGTATGGGTCAGGCACCGAGCAATGCTGTAGCGGTGTTTCGTCCTTTCCACAA |
| LRRK2 P14_10C | 5’ AAAAAAACTACAGCAGTGCTCAGTATGGGTCAGGCACCGAGCAATGCTGTAGCGGTGTTTCGTCCTTTCCACAA |
| LRRK2 P14_15A | 5’ AAAAAAACTATAGCATTGCTCAGTATGGGTCAGGCACCGAGCAATGCTGTAGCGGTGTTTCGTCCTTTCCACAA |
| LRRK2 P14_15C | 5’ AAAAAAACTAAAGCATTGCTCAGTATGGGTCAGGCACCGAGCAATGCTGTAGCGGTGTTTCGTCCTTTCCACAA |
| LRRK2 P15_16A | 5’ AAAAAACTATAGCATTGCTCAGTACTGGGTCAGGATACCGAGCAATGCTGTACGGTGTTTCGTCCTTTCCACAA |
| LRRK2 P15_16C | 5’ AAAAAACTAAAGCATTGCTCAGTACTGGGTCAGGATACCGAGCAATGCTGTACGGTGTTTCGTCCTTTCCACAA |
| LRRK2 P11_9A | 5’ AAAAAACTGACTACAGTATTGCTCATGGGTCAGGCGAACAATACTGTAGTCACGGTGTTTCGTCCTTTCCACAA |
| LRRK2 P11_9C | 5’ AAAAAACTGACTACAGGATTGCTCATGGGTCAGGCGAACAATCCTGTAGTCACGGTGTTTCGTCCTTTCCACAA |
| LRRK2 P11_9U | 5’ AAAAAACTGACTACAGAATTGCTCATGGGTCAGGCGAACAATTCTGTAGTCACGGTGTTTCGTCCTTTCCACAA |
| LRRK2 P11_10U | 5’ AAAAAACTGACTACAACATTGCTCATGGGTCAGGCGAACAATGCTGTAGTCACGGTGTTTCGTCCTTTCCACAA |
| LRRK2 P11_10A | 5’ AAAAAACTGACTACATCATTGCTCATGGGTCAGGCGAACAATGCTGTAGTCACGGTGTTTCGTCCTTTCCACAA |
| LRRK2 P11_10G | 5’ AAAAAACTGACTACACCATTGCTCATGGGTCAGGCGAACAATGGTGTAGTCACGGTGTTTCGTCCTTTCCACAA |
|  | **psiCheck targets** |
| LRRK2 WT PSICHECK + | 5’ TCGAGATATCGCCATCATTGCAAAGATTGCTGACTACGGCATTGCTCAGTACTGCTGTAGAAGC |
| LRRK2 WT PSICHECK - | 5’ GGCCGCTTCTACAGCAGTACTGAGCAATGCCGTAGTCAGCAATCTTTGCAATGATGGCGATATC |
| LRRK2 MT PSICHECK + | 5’ TCGAGATATCGCCATCATTGCAAAGATTGCTGACTACAGCATTGCTCAGTACTGCTGTAGAAGC |
| LRRK2 MT PSICHECK - | 5’ GGCCGCTTCTACAGCAGTACTGAGCAATGCTGTAGTCAGCAATCTTTGCAATGATGGCGATATC |

**Table S1: Oligonucleotide sequences used**
